# Complete Genome Sequence of Bovine Coronavirus in Blood Diarrhea from Adult Cattle That Died from Winter Dysentery in Japan

**DOI:** 10.1128/MRA.00807-21

**Published:** 2021-10-21

**Authors:** Keisuke Nakagawa, Hitomi Kumano, Yuko Kitamura, Keisuke Kuwata, Eiji Tanaka, Hideto Fukushi

**Affiliations:** a Joint Graduate School of Veterinary Sciences, Gifu University, Yanagido, Gifu, Japan; b Laboratory of Veterinary Microbiology, Joint Department of Veterinary Medicine, Gifu University, Yanagido, Gifu, Japan; c Education and Research Center for Food Animal Health, Gifu University, Yanagido, Gifu, Japan; d Gifu Prefectural Chuo Livestock Hygiene Service Center, Yanagido, Gifu, Japan; e The United Graduate School of Veterinary Sciences, Gifu University, Yanagido, Gifu, Japan; KU Leuven

## Abstract

We determined the complete genome sequence of bovine coronavirus (BCoV) recovered from bloody diarrhea from adult cattle that died from winter dysentery in 2020 in Japan. Information on the complete genome sequence of BCoV, which causes deadly diarrhea in adult cattle, has great potential for a better understanding of its pathogenicity.

## ANNOUNCEMENT

Bovine coronavirus (BCoV) is responsible for respiratory disease and winter dysentery in cattle (1). BCoV belongs to the order *Nidovirales*, family *Coronaviridae*, and the genus *Betacoronavirus*. BCoV consists of a nonsegmented, single-stranded, positive-sense RNA (2). Zhang et al. showed that in the process of cell culture adaptation, an enteric BCoV strain accumulated mutations to resemble the corresponding respiratory BCoV isolate from the same animal (3), suggesting the importance of sequencing of the enteric BCoV genome without laboratory manipulation for a better understanding of the pathogenesis of enteric BCoV. We determined the complete genome sequence of a BCoV strain, termed GF2020, recovered from bloody diarrhea from adult cattle that died from winter dysentery in 2020 in Gifu Prefecture, Japan, without virus isolation in cell culture.

RNA was extracted from the bloody diarrhea using a Direct-zol RNA miniprep kit (Zymo Research). cDNA was synthesized using SuperScript III reverse transcriptase (Invitrogen) and random primers (Invitrogen). The whole viral genome, comprising six overlapping amplicons, except for 70 nucleotides (nt) at the 5′ end and 97 nt at the 3′ end, was generated from cDNA using KOD FX polymerase (Toyobo) and the sets of primers shown in Table 1. From an equimolar mixture of 6 PCR products, sequencing libraries were prepared using the NEBNext Ultra II DNA library prep kit (NEB). The libraries were sequenced on a NovaSeq 6000 instrument (Illumina). A paired-end sequencing run with a 2 × 150-nucleotide read length generated 9,654,684 raw reads. The raw reads were trimmed using Trimmomatic (4) and mapped to BCoV reference genome strain TCG-27 (GenBank accession number LC494186.1) using Bowtie2 (5). All tools were run with default parameters unless otherwise specified.

**TABLE 1:** Primers used in this study

Primer^*a*^	Sequence (5′ to 3′)	Nucleotide position
RT-PCR		
PCR1 F	GTTAGATCTTTTCATAATCTAAAC	47–70
PCR1 R	CATTTCCTAGACTCTTACCAAAACTT	4962–4987
PCR2 F	GTTCAGAGCAATGTTGATGTTGTA	4696–4719
PCR2 R	AAGAATGAAGTTGAAACAGAAGCAGT	9916–9941
PCR3 F	AGTGTGATAATGCATTTACAATGGCT	9526–9551
PCR3 R	AAAATTTAGTGGTGCCTATAACAA	15061–15084
PCR4 F	ACTAAGCGCAATGTCCTGCCAACA	14892–14915
PCR4 R	CTCAGGCTGTCGAATTGGCTGAAG	20018–20041
PCR5 F	GATTATGCTAGAGAAAGTATATTTTG	19728–19753
PCR5 R	TGTTGTGCATAAACAACATCATGA	25047–25070
PCR6 F	CCGACGTATACCTAACCTTCCCGATTGC	24603–24630
PCR6 R	CTTCCCCTTGGGCACTTGTCGGCA	30919–30942
3′-terminal sequencing		
Anchored oligo(dT) primer	GTCGTGACTGGGAAAACTTTTTTTTTTTTTTTTTTTT	Poly(A)
F 3′-terminal seq 1	CAATCAGAATTTTGGTGGTG	30252–30271
F 3′-terminal seq 2	TCAGGTTTTGAGACCATAATG	30469–30489
R 3′ anker	GTCGTGACTGGGAAAAC	Anker sequence
5′ RACE		
5′ phosphorylated RT primer	CATAAGCCTCTAAGC	1010–1024
5′ RACE S1	ATGTCTCTGGAGGCATGCTAT	541–561
5′ RACE S2	GACCATGGGTTTGTTTCGGC	588–607
5′ RACE R1	CCACCTCTGAACTACTAGGGTTG	297–319
5′ RACE R2	TGCGTCCTCAAACATCCATG	263–282

a F, forward; R, reverse.

The 5′-terminal sequence of the genomic RNA of strain GF2020 was determined using the rapid amplification of cDNA ends (RACE) method (5′-full RACE core set; TaKaRa Bio). The 3′ end of the viral genome was amplified using specific primers and anchored oligo(dT) primers. Information about the primers is provided in Table 1. The amplicon containing the 5′- and 3′-terminal sequences of the viral genome was sequenced using a BigDye Terminator v3.1 cycle sequencing kit (Applied Biosystems) on an ABI PRISM 3100 DNA analyzer (Applied Biosystems).

**FIG 1:**
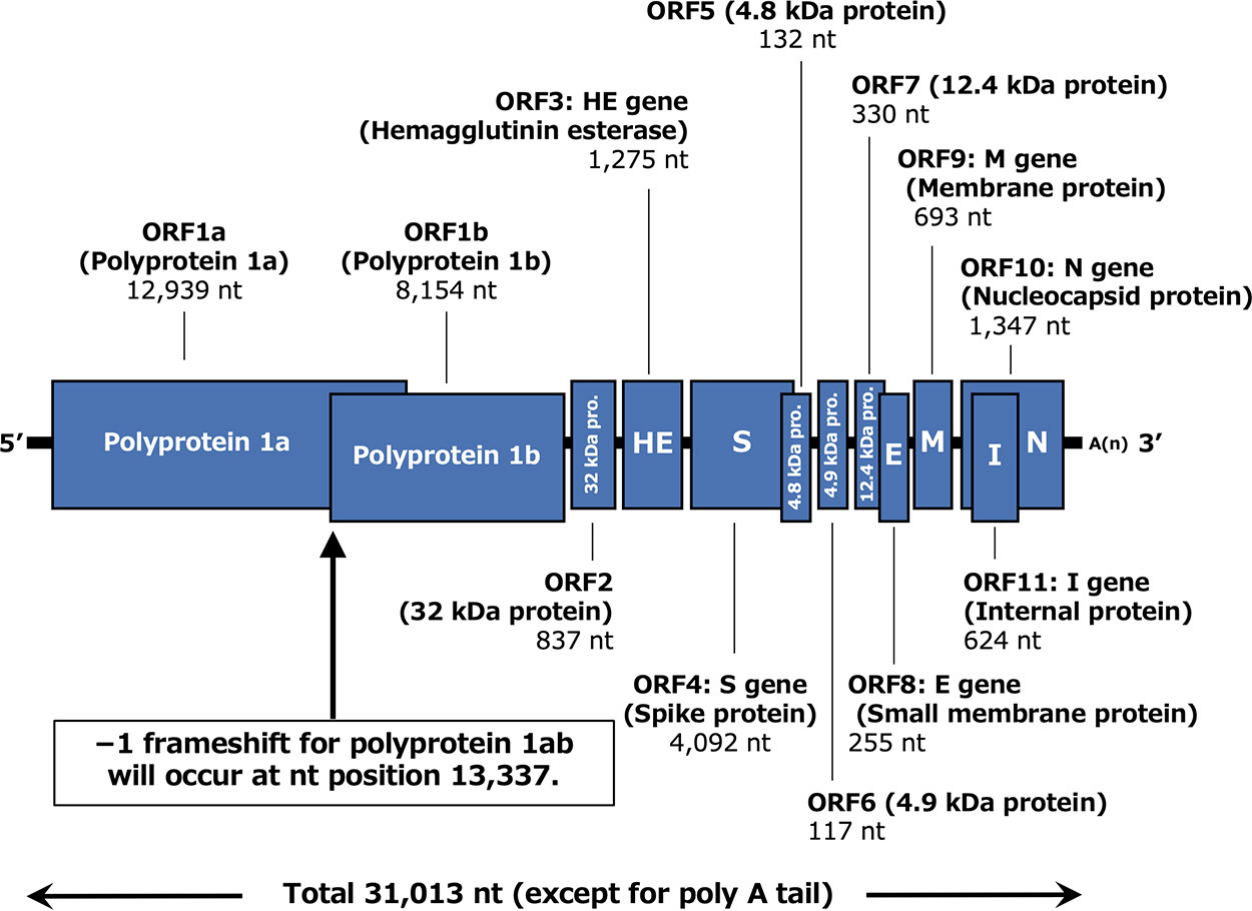
Genome characteristics determined for BCoV strain GF2020 in the current study. The nucleotide lengths and coding proteins for ORF1a, ORF1b, and ORF2-ORF11 are shown. The 5′ and 3′ untranslated regions (bars) and viral open reading frames (boxes) are not drawn to scale.

The determined sequences were visually assembled using ApE (https://jorgensen.biology.utah.edu/wayned/ape/) with the NCBI reference sequence of isolate BCoV-ENT (GenBank accession number NC_003045.1), resulting in the following genome organization (Fig. 1): 5′ untranslated region (UTR)-ORF1a (polyprotein 1a)-ORF1b (polyprotein 1b)-ORF2 (32 kDa protein)-ORF3 (hemagglutinin esterase)-ORF4 (spike protein)-ORF5 (4.9 kDa protein)-ORF6 (4.8 kDa protein)-ORF7 (12.4 kDa protein)-ORF8 (small membrane protein)-ORF9 (membrane protein)-ORF10 (nucleocapsid protein)-ORF11 (internal protein)-3′ UTR. The full-length genome sequence of strain GF2020 comprised 31,013 nucleotides with a G+C content of 37.0%. According to BLASTN analysis (6), the full-length genome sequence showed the highest nucleotide identity (99.44%) with BCoV strain TCG-27 (GenBank accession number LC494186.1), which was collected from a nasal swab from a dairy calf in Japan (7), supporting the notion that no genetic markers have been identified to discriminate BCoV from respiratory and enteric syndromes (1, 7). Accumulation of information on the whole viral genome of enteric BCoV without laboratory manipulation will contribute to a better understanding of the virulence factors involved in BCoV-mediated winter dysentery in cattle.

### Data availability.

The complete genome sequence of strain GF2020 has been deposited in GenBank under the accession number LC642814. The raw data were deposited under SRA accession number DRX301428, BioSample accession number SAMD00393674, and BioProject accession number PRJDB12037.
